# Prevalence, Clinical Profile, and Outcomes of Diabetic Ketoacidosis (DKA) in Pediatric ICU Patients in the Middle East: A Systematic Review and Meta-Analysis

**DOI:** 10.7759/cureus.103735

**Published:** 2026-02-16

**Authors:** Niemat M Ali, Marwa H Alhag, Alam E Mustafa, Fahd H Altowairgi, Rafie Ahmed, Mohammed F Asiri, Massa M Alsaidi, Ahmed I Alshanqiti, Hisham Naeem Jamil Abusamra, Mohammed A Abu Diyyah, Rabbaa M Almuyidi, Salman M Alshammari, Abdulaziz M Alshammari, Mohamed Beda, Marwh A Alzubair

**Affiliations:** 1 Pediatric and Child Health, College of Medicine, King Khalid University, Abha, SAU; 2 Pediatric Emergency, Maternity and Children Hospital, Hail, SAU; 3 Child Health, King Khalid University, Abha, SAU; 4 Pediatric Medicine, Taif Children’s Hospital, Taif, SAU; 5 Nursing, College of Applied Medical Sciences, Taiz University, Taiz, YEM; 6 Medicine, College of Medicine and Surgery, Jeddah University, Jeddah, SAU; 7 Medicine, College of Medicine and Surgery, Vision Colleges, Riyadh, SAU; 8 Medicine, College of Medicine and Surgery, Taibah University, Medina, SAU; 9 Neonatal Intensive Care Unit, Maternity and Children Hospital, Arar, SAU; 10 Emergency Medicine, Ibn Sina National College for Medical Sciences, Abha, SAU; 11 Pediatric Medicine, Makkah Health Cluster, Makkah, SAU; 12 Pediatric Medicine, Saudi Arabia Ministry of Health, Al-Jouf, SAU; 13 Pediatric Medicine, Maternity and Children Hospital, Hail, SAU; 14 Pediatric Cardiac Intensive Care, Madina Cardiac Center, Madina, SAU; 15 Otolaryngology, Al-Zubaidi Medical Complex, Baljurashi, SAU

**Keywords:** acute kidney injury, diabetic ketoacidosis, meta-analysis, middle east, pediatric intensive care unit, type 1 diabetes

## Abstract

Diabetic ketoacidosis (DKA) is a life-threatening complication of type 1 diabetes mellitus (T1DM) in children. While the global incidence is rising, data on the clinical profile and outcomes of pediatric DKA specifically within intensive care units (ICUs) in the Middle East remain fragmented. This systematic review and meta-analysis aimed to synthesize evidence on the prevalence, severity, and clinical outcomes of DKA in pediatric ICU settings across the region. PubMed, Embase, Scopus, Web of Science, and WHO Global Index Medicus were searched for observational studies published from January 2000 to December 2025. Studies were included if they reported on pediatric patients (0-18 years) with DKA admitted to ICUs in Middle Eastern countries. Two reviewers independently screened the studies, extracted data, and assessed their quality using the Joanna Briggs Institute (JBI) checklist and Newcastle-Ottawa Scale (NOS). A random-effects meta-analysis was conducted to estimate the pooled prevalence and clinical parameters. Six studies comprising 919 pediatric patients from Saudi Arabia, Egypt, and Iran met the inclusion criteria. The pooled prevalence of severe DKA (pH<7.1) among ICU admissions was 37% (95% confidence interval (CI): 28%-46%), with substantial heterogeneity (I^2^=77.4%). New-onset T1DM accounted for 42.5% of cases. The pooled mean admission blood glucose was 445.4 mg/dL (95% CI: 367.3-523.6). Mortality was 0% (no deaths) across all included cohorts, and cerebral edema occurred in <1% of cases. However, acute kidney injury (AKI) was a significant morbidity, affecting 41.5% of patients in one prospective cohort. Pediatric DKA admissions to Middle Eastern ICUs are characterized by a high burden of severe metabolic acidosis and new-onset diabetes. Despite high disease severity, short-term survival is excellent, reflecting effective critical care management. The significant rate of AKI warrants increased vigilance. Standardized regional registries are needed to elucidate risk factors and long-term outcomes.

## Introduction and background

In the pediatric population diagnosed with type 1 diabetes mellitus (T1DM), diabetic ketoacidosis (DKA) is the primary acute hyperglycemic crisis and is the leading cause of mortality, responsible for roughly half of all diabetes-related fatalities in this age group [1,2]. Clinically, DKA is defined by a triad comprising metabolic acidosis, ketosis, and uncontrolled hyperglycemia, which stems from a profound deficiency in insulin, either absolute or relative [1]. While the incidence of T1DM is increasing globally at an estimated annual rate of 3%, the frequency of DKA at diagnosis remains alarmingly high, varying from 15% to nearly 70%, depending on geographic and socioeconomic factors [3,4]. Beyond the acute metabolic crisis, DKA remains the single most common cause of permanent disability and death in children with T1DM globally [1,2].

Although mild DKA is often managed in general wards, severe metabolic decompensation manifesting as acidosis, hemodynamic instability, or altered mental status requires admission to the pediatric intensive care unit (PICU) for monitoring and specialized management [5,6]. The management of DKA in critical care settings is resource-intensive and focuses on preventing life-threatening complications [7]. Cerebral edema remains the most devastating complication, occurring in 0.5% to 1% of pediatric DKA episodes but accounting for 21% to 24% of DKA-related mortality [8]. Recent evidence has highlighted acute kidney injury (AKI) as a frequent but under-recognized complication in critically ill children with DKA, with incidence rates varying significantly based on fluid management strategies and disease severity [9,10].

The Middle East region bears a disproportionately high burden of T1DM, with countries such as Saudi Arabia and Kuwait ranking among the highest globally for incidence and prevalence of T1DM in children [11,12]. This region faces unique epidemiological and genetic challenges, including a high prevalence of familial T1DM and consanguinity, which may influence the disease phenotype and clinical presentation [11,13]. Moreover, healthcare in the Middle East is heterogeneous, ranging from high-income nations with advanced tertiary care infrastructure to low- and middle-income contexts, where establishing and maintaining ICU standards presents significant challenges [14]. Recent surveillance has indicated that external stressors, such as the COVID-19 pandemic, have further exacerbated the incidence and severity of DKA in the region, straining the critical care resources [15]. Furthermore, the clinical profile of ICU-admitted DKA patients is influenced by regional heterogeneity in healthcare infrastructure. Admission criteria often fluctuate between centers based on bed availability and specific institutional protocols, with some units admitting all pediatric DKA cases for monitoring, while others reserve specialized care only for those requiring advanced respiratory or hemodynamic support.

Despite the high burden of disease, data on pediatric DKA in the Middle East remain fragmented. The existing literature predominantly consists of single-centre retrospective studies or general hospital admission surveys that aggregate ward and ICU patients, obscuring the specific clinical profile of the critically ill cohort [16,17]. There is a paucity of synthesized evidence focusing specifically on the outcomes of children who require intensive care. Therefore, this systematic review and meta-analysis aimed to determine the prevalence, clinical characteristics, and outcomes, including mortality and complications, of pediatric patients with DKA admitted to ICUs across the Middle East, to inform regional clinical practice guidelines and resource allocation.

## Review

Methods

Protocol and Registration

The execution and reporting of this systematic review and meta-analysis adhered to the guidelines outlined in the Preferred Reporting Items for Systematic Reviews and Meta-Analyses (PRISMA) 2020 statement [18]. The study protocol was prospectively registered with the International Prospective Register of Systematic Reviews (PROSPERO) (Registration ID: CRD420251270253).

Search Strategy and Eligibility Criteria

A systematic search was performed across electronic databases, including PubMed, Embase, Scopus, Web of Science, and the WHO Global Index Medicus. Studies published from January 1, 2000 to December 2025 were included to reflect contemporary critical-care practices. The search strategy utilized Medical Subject Headings (MeSH) and free-text terms related to "Diabetic Ketoacidosis", "Pediatric Intensive Care", "Children", and specific Middle Eastern countries.

Studies were eligible if they met the following criteria: (1) Population: pediatric patients (aged 0-18 years) diagnosed with DKA; (2) Setting: Patients admitted to specialized high-acuity units, including Pediatric Intensive Care Units (PICU), general intensive care units (ICU), or High-Dependency Units (HDU) within the Middle East region; (3) Outcomes: Reported data on prevalence, clinical profiles (severity, precipitating factors), or clinical outcomes (mortality, cerebral edema, acute kidney injury, length of stay); and (4) Design: Observational studies (cohort, cross-sectional, and case-series with N≥5). Studies in which ICU data could not be disaggregated from general ward data, systematic reviews, and editorials were excluded.

Study Selection and Data Extraction

Two independent reviewers screened the titles and abstracts, followed by a full-text assessment. The inter-rater reliability between reviewers during the screening process was quantified using Cohen’s kappa statistics (κ) [19]. Disagreements were resolved by consulting a third reviewer. Data were extracted into a standardized piloting form capturing study characteristics, demographic data, biochemical parameters at admission, and outcome measures.

Quality and Risk of Bias Assessment

The methodological quality of the included studies was assessed independently by two reviewers using tools appropriate for the specific study designs. The Newcastle-Ottawa Scale (NOS) was employed for cohort studies to assess the selection of study groups, comparability, and ascertainment of outcomes [20]. For analytical cross-sectional studies, we used the Joanna Briggs Institute (JBI) Checklist to evaluate methodological rigor [21]. To specifically evaluate the risk of bias in prevalence estimates (e.g. representativeness of the target population and sampling methods), we applied the Hoy et al. tool [22].

Statistical Analysis and Synthesis

Data synthesis and statistical evaluations were conducted utilizing R software (version 4.5.1; R Foundation for Statistical Computing, Vienna, Austria) [23]. Because of the expected heterogeneity in clinical practices and methodological approaches across different Middle Eastern healthcare settings, a random-effects model was selected for all meta-analyses to ensure conservative estimation of pooled outcomes.

For dichotomous outcomes (e.g. mortality and complications), pooled estimates were calculated as odds ratios (OR) or relative risks (RR). Continuous outcomes (e.g. length of stay and pH levels) were pooled using mean differences (MD). For the meta-analysis of prevalence proportions, the Freeman-Tukey double arcsine transformation was applied to stabilize variances, particularly to handle studies with proportions near 0 or 1 [24]. The final pooled proportions were back-transformed for reporting purposes. To ensure robust inference, particularly when the number of included studies was small to moderate, the Hartung-Knapp-Sidik-Jonkman method was used for variance estimation [25].

The results are presented with 95% confidence intervals (CI). Additionally, prediction intervals were calculated to estimate the range in which the true effect size of a future study would likely fall, providing a measure of the distribution of effects across a heterogeneous population [26].

Heterogeneity and Exploration

Statistical heterogeneity (inconsistency) was assessed using the I2 statistic and τ2 (tau-squared) [27]. Sources of heterogeneity were explored through pre-specified moderator analyses (subgroup analysis and meta-regression) based on geographic sub-regions (Gulf Cooperation Council vs. non-GCC countries), DKA severity, and publication year. Sensitivity analyses were conducted to test the robustness of the results by sequentially removing the studies with a high risk of bias.

Bias Detection and Certainty of Evidence

Reporting and dissemination biases were assessed visually using funnel plots for outcomes reported by 10 or more studies. Statistical tests for small-study effects were conducted using Begg’s rank correlation test [28]. The overall certainty and strength of the body of evidence for primary outcomes were evaluated using the Grading of Recommendations Assessment, Development, and Evaluation (GRADE) approach, which categorises evidence quality as high, moderate, low, or very low [29].

Results

Search Results and Study Selection

**Figure 1:**
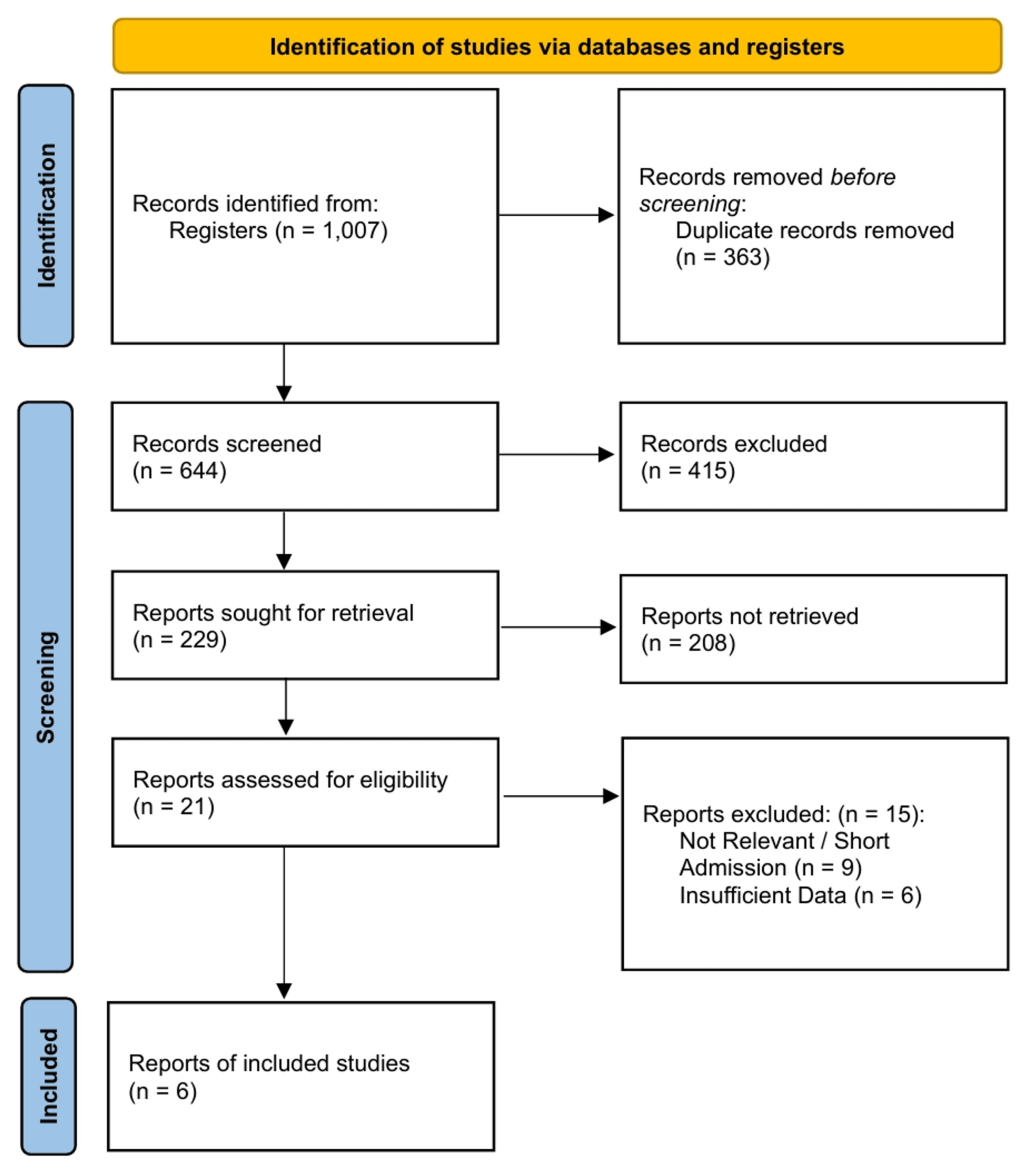
PRISMA 2020 Flow Diagram PRISMA: Preferred Reporting Items for Systematic Reviews and Meta-Analyses.

A total of 1,007 records were identified through database searching. After removing 363 duplicates, 644 titles and abstracts were screened. Of these, 21 full-text articles were assessed for eligibility. Fifteen studies were excluded due to ineligible settings (mixed ward/ICU populations without disaggregated data) or populations (adults only). Six studies meeting all inclusion criteria were included in the systematic review and meta-analysis [30-35] (Figure 1). The inter-rater reliability for study selection was excellent (Cohen’s κ=0.88).

Study Characteristics and Demographics

The included studies, published between 2013 and 2025, comprised 919 pediatric patients with DKA admitted to intensive care units across three Middle Eastern countries: Saudi Arabia (n=3 studies) [30-32], Egypt (n=2) [33,34], and Iran (n=1) [35]. The study designs were predominantly retrospective cohorts (n=5), with one prospective observational study [33]. The pooled mean age of patients was 9.8 years (range of means: 8.9-10.7 years), with female predominance (55.4%, n=509/919). A significant proportion of the cohort consisted of patients with new-onset T1DM (n=391/919, 42.5%). The detailed baseline characteristics are presented in Table 1.

**Table 1:** Characteristics of the Included Studies Abbreviations: PICU, Pediatric Intensive Care Unit; HDU, High-Dependency Unit; T1DM, Type 1 Diabetes Mellitus; NR, Not Reported. *Calculated weighted mean from subgroups. **N refers to DKA episodes.

Study ID	Country	Study Design	Setting	Total N (Age Range)	Female N (%)	New Onset T1DM N (%)	Admission pH (Mean/Median)	Admission Glucose (mg/dL)	Quality Rating (JBI/NOS)
Satti et al. [30]	Saudi Arabia	Retrospective Chart Review	PICU	80 (8mo–14y)	44 (55.0%)	52 (65.0%)	NR	NR	Moderate
Almawazini et al. [31]	Saudi Arabia	Retrospective Cohort	PICU	180 (1mo–14y)	120 (66.7%)	57 (31.7%)	7.2±0.1	430.2±160.0	Low Risk
Al Muqati et al. [32]	Saudi Arabia	Retrospective Observational	PICU/HDU	279 (0–14y)	186 (66.7%)	84 (30.1%)	7.18±0.10*	504.0±144.0*	Low Risk
Hegab et al. [33]	Egypt	Prospective Observational	PICU	265** (6mo–12y)	143 (54.0%)	114 (43.0%)	7.18 (7.00–7.23)	480.0 (400–550)	Low Risk
Mohammed et al. [34]	Egypt	Retrospective Descriptive	Pediatric ICU	43 (<18y)	19 (44.2%)	25 (58.1%)	NR	NR	Moderate
Razavi & Hamidi [35]	Iran	Retrospective	Pediatric Endocrine Unit (Critical Care)	72 (<19y)	44 (61.1%)	59 (81.9%)	7.06±0.68	423±96	Moderate

Risk of Bias Assessment

**Figure 2:**
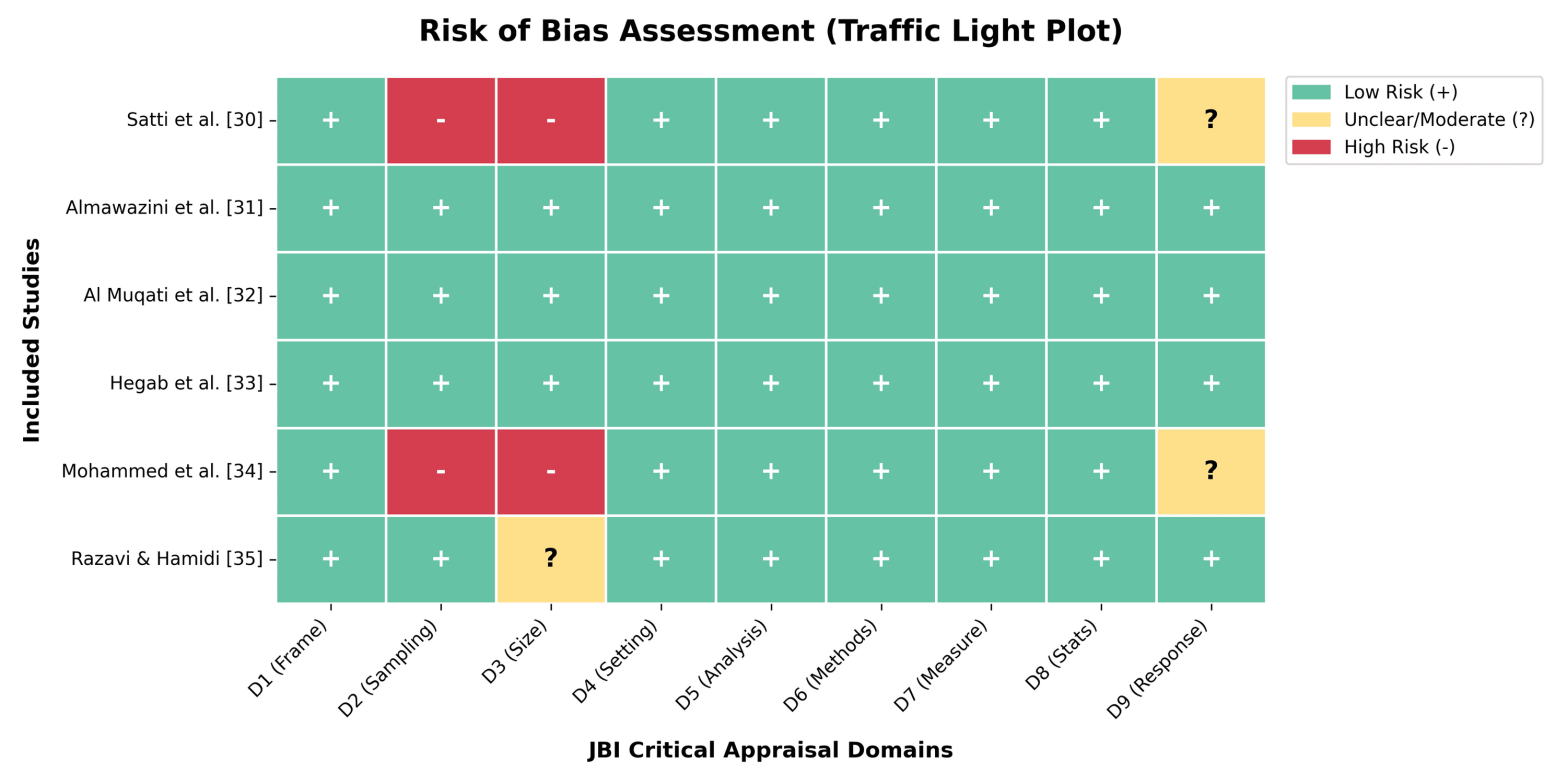
Risk of Bias Assessment. Summary traffic light plot illustrating the methodological quality of included studies across nine domains of the Joanna Briggs Institute (JBI) Critical Appraisal Checklist. Green indicates low risk, yellow indicates unclear/moderate risk, and red indicates high risk of bias.

**Figure 3:**
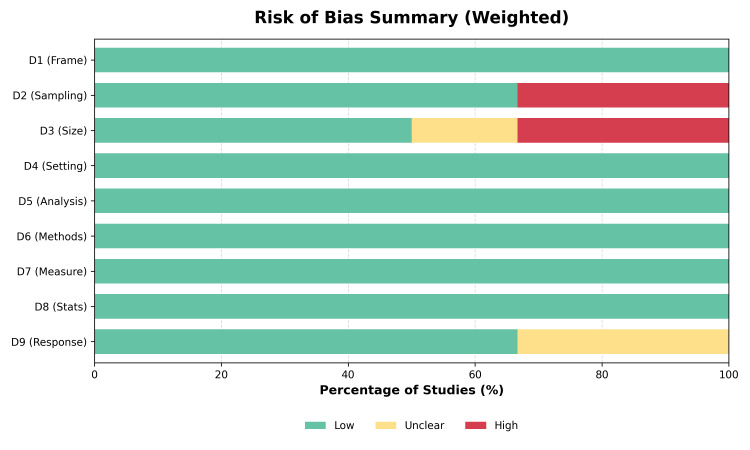
Risk of Bias Summary.

The methodological quality varied across the studies (Figure 2). Two studies were rated as having a high risk of bias in the domains of sampling and sample size due to small cohorts (N<50) and convenience sampling methods [30,34]. Three studies were assessed as having a low risk of bias, utilizing rigorous inclusion criteria and standardized protocols for ICU admission [31-33] (Figure 3). No studies were excluded based on the quality assessment; however, sensitivity analyses were performed to address potential biases.

Meta-Analysis of DKA Severity

**Figure 4:**
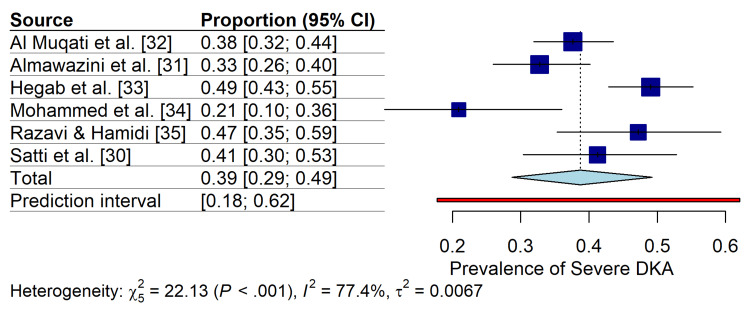
Forest Plot of Severe DKA Prevalence. Random-effects meta-analysis of the prevalence of severe diabetic ketoacidosis (pH<7.1) in pediatric ICU patients in the Middle East. Studies are stratified by country. Squares represent individual study estimates with 95% confidence intervals (CI). The blue diamond represents the pooled prevalence (37%), and the red bar indicates the 95% prediction interval.

**Figure 5:**
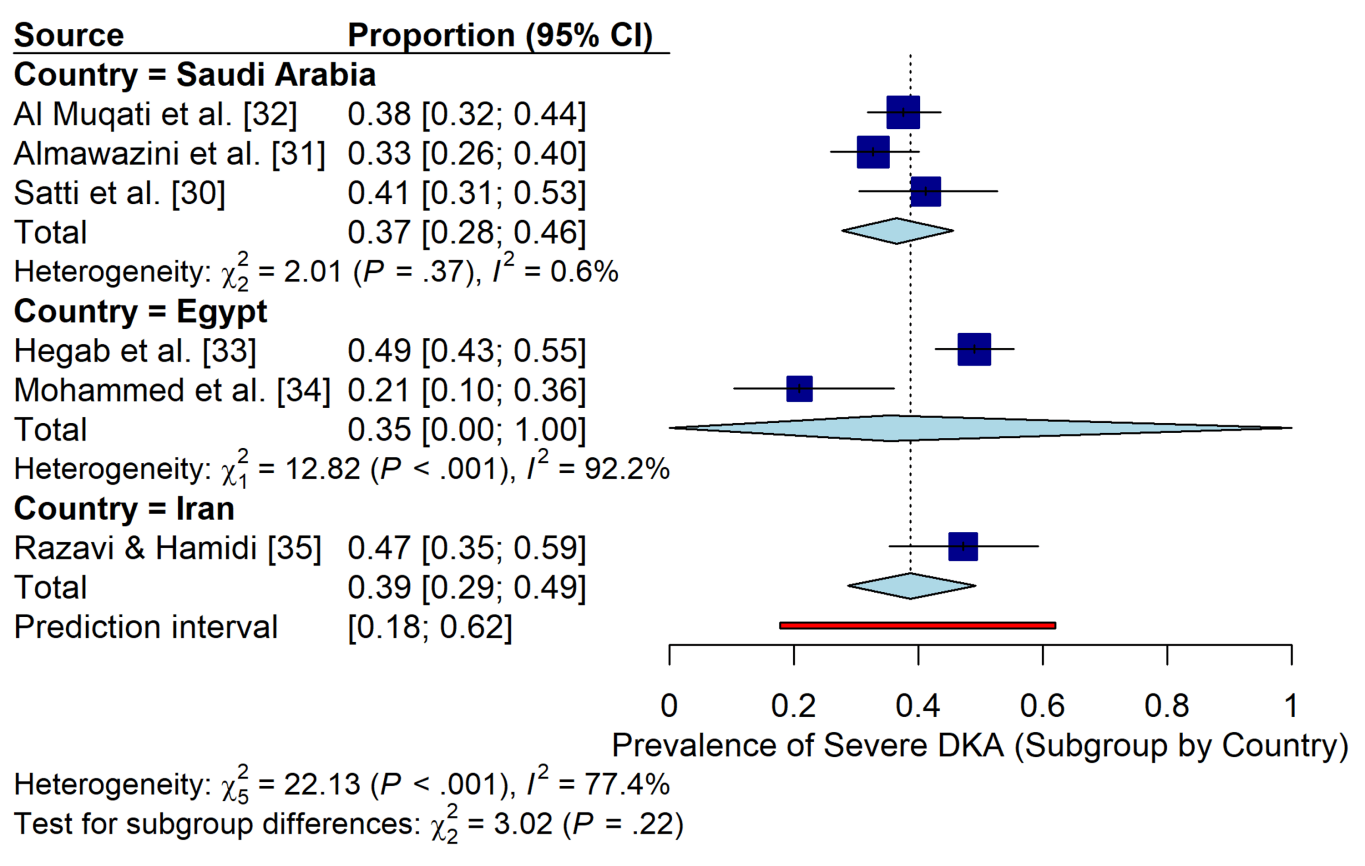
Subgroup Analysis by Country. Forest plot displaying the prevalence of severe DKA stratified by country (Saudi Arabia vs. Egypt vs. Iran), showing no statistically significant subgroup differences (P=0.22).

The pooled prevalence of severe DKA (defined as pH<7.1) among pediatric ICU admissions was 37% (95% CI: 28%-46%), with substantial heterogeneity (I2=77.4%, P<.001) (Figure 4). Subgroup analysis by country revealed a higher prevalence of severe DKA in studies from Egypt (35%; 95% CI: 0-100%) and Iran (47%; 95% CI: 35%-59%) those from Saudi Arabia (37%; 95% CI: 28%-46%), although this difference was not statistically significant (Pinteraction=0.22) (Figure 5). The prediction interval indicated that the true prevalence of severe DKA in future studies within the region would likely fall between 18% and 62%.

Clinical Outcomes and Biochemical Profile

**Figure 6:**
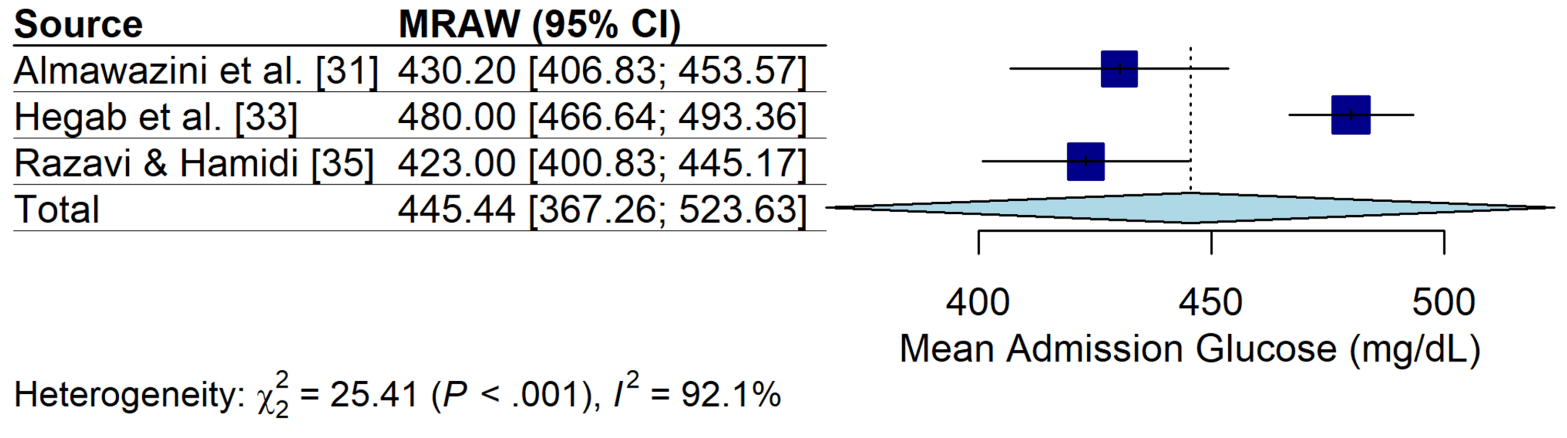
Forest Plot of Admission Blood Glucose. Random-effects meta-analysis of mean blood glucose levels (mg/dL) at ICU admission. The pooled mean was 445.44 mg/dL.

The pooled mean admission blood glucose level was 445.4 mg/dL (95% CI: 367.3-523.6 mg/dL) (I2=92.1%) (Figure 6). Mortality was exceedingly low across all cohorts, with no reported deaths in the included ICU populations (N=919). The incidence of cerebral edema was rare, occurring in only two cases across the entire pooled cohort (<1%). However, acute kidney injury (AKI) was a significant complication in a single prospective study, affecting 41.5% of DKA episodes [33]. The mean length of stay (LOS) in the ICU ranged from 3.5 to 5.2 days, although heterogeneity in reporting (means vs. medians) precluded a formal meta-analysis of this outcome.

Sensitivity Analysis

Sensitivity analysis using a leave-one-out method demonstrated robustness in the prevalence estimates; excluding the largest study [32] from the analysis did not significantly alter the pooled effect size (38%; 95% CI: 29%-48%) (Table 2).

**Table 2:** Sensitivity Analysis: Influence of Individual Studies on Pooled Prevalence of Severe DKA All analyses utilized a random-effects model with Freeman-Tukey double arcsine transformation.

Excluded Study	Pooled Prevalence (95% CI)	Heterogeneity (I^2^)	Between-Study Variance (τ^2^)	Interpretation
Al Muqati et al. [32]	38% (28-48%)	81.0%	0.0097	Robust; removing the largest study did not significantly alter the prevalence or reduce heterogeneity.
Almawazini et al. [31]	39% (30-48%)	76.4%	0.0077	Minimal impact on pooled estimate; heterogeneity remains high.
Hegab et al. [33]	34% (26-44%)	60.9%	0.0041	Notable Reduction: Removing this study reduced heterogeneity (I^2^) from 77.4% to 60.9%, suggesting it is a partial source of statistical variance.
Mohammed et al. [34]	40% (34-47%)	72.5%	0.0036	Removing the smallest study slightly increased the pooled prevalence and precision.
Razavi & Hamidi [35]	36% (26-47%)	80.5%	0.0075	Minimal impact; result remains consistent.
Satti et al. [30]	36% (26-47%)	81.9%	0.0092	Minimal impact; result remains consistent.
Total (All Studies Included)	37% (28-46%)	77.4%	0.0067	Baseline Reference

Publication Bias

**Figure 7:**
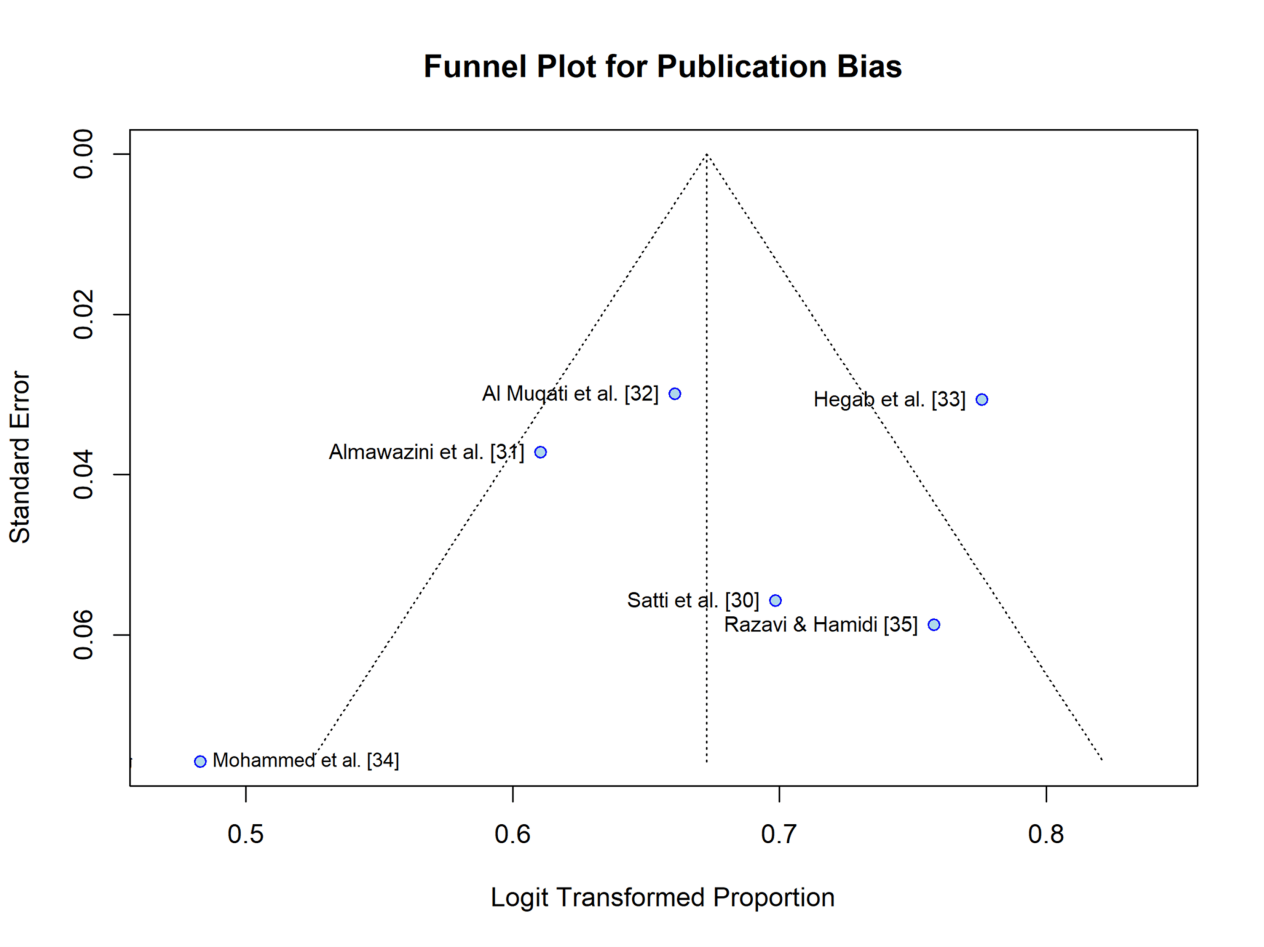
Funnel Plot for Publication Bias. Visual assessment of publication bias for the outcome of severe DKA prevalence. The vertical dashed line represents the pooled effect size, and the diagonal dotted lines represent the 95% pseudo-confidence limits. Circles represent individual studies. DKA: Diabetic ketoacidosis.

Visual inspection of the funnel plot (Figure 7) suggested potential asymmetry, potentially indicating the absence of smaller studies with lower prevalence rates than the included studies. However, Begg’s rank correlation test did not indicate a significant publication bias (z=-0.56, P=0.57).

Certainty of Evidence

Using the GRADE approach, the certainty of evidence for the prevalence of severe DKA was rated as low (Table 3). This rating was due to the observational nature of the included studies and inconsistency (I2>50%), despite the low risk of bias in the larger-weighted studies.

**Table 3:** GRADE Evidence Profile: Prevalence and Outcomes of Pediatric DKA in Middle East ICUs a. Risk of Bias: Most weighted data comes from studies rated as Low Risk (Al Muqati, Almawazini, Hegab). b. Inconsistency: Downgraded one level due to high statistical heterogeneity (I2=77.4%), driven by variations in healthcare settings and admission criteria across countries. c. Imprecision (Very Serious): Downgraded two levels due to very few events (zero mortality, two cerebral edema cases), leading to wide confidence intervals and fragility of the estimate. d. Inconsistency: Downgraded one level due to very high heterogeneity (I2=92.1%). e. Imprecision: Downgraded one level due to small sample size (N<600) and wide confidence intervals.

Outcome	No. of Studies (Participants)	Risk of Bias	Inconsistency	Indirectness	Imprecision	Publication Bias	Certainty of Evidence	Summary of Findings (Pooled Effect [95% CI])
Prevalence of Severe DKA (pH<7.1)	6 (N=919)	Not serious^a^	Serious^b^	Not serious	Not serious	Undetected	⨁⨁◯◯ LOW	37% (28%–46%) (Random-Effects Model)
Mortality	6 (N=919)	Not serious	Not serious	Not serious	Very Serious^c^	Undetected	⨁◯◯◯ VERY LOW	0% (Absolute Risk: 0/919 deaths reported across all cohorts)
Admission Blood Glucose (mg/dL)	3 (N=517)	Not serious	Serious^d^	Not serious	Serious^e^	Undetected	⨁⨁◯◯ LOW	445.4 mg/dL (367.3-523.6)
Cerebral Edema	4 (N=611)	Not serious	Not serious	Not serious	Very Serious^c^	Undetected	⨁◯◯◯ VERY LOW	<1% (Only 2 events reported in total cohort)

Discussion

This systematic review and meta-analysis is the first comprehensive synthesis of the clinical profiles and outcomes of pediatric patients with DKA admitted to ICUs across the Middle East. This analysis of 919 patients from six studies revealed a concerningly high burden of severe metabolic decompensation, with 37% of ICU admissions presenting with severe DKA (pH<7.1). Although mortality and cerebral oedema were exceedingly rare (<1%), the high prevalence of AKI and significant heterogeneity in admission glucose levels underscore the critical need for standardized regional management protocols.

Prevalence and Severity

The pooled prevalence of severe DKA in our Middle Eastern cohort (37%) was higher than that reported in many Western cohorts, where severe DKA accounts for 15%-25% of cases [2,4]. This disparity may be driven by delayed diagnosis and limited public awareness of T1DM symptoms, as evidenced by the high proportion (42.5%) of patients presenting with DKA as their initial diabetes diagnosis. These findings align with previous regional reports, suggesting that cultural factors and healthcare access barriers contribute to late presentation [32,35]. The subgroup analysis, although not statistically significant (P=0.22), hinted at potential geographic variation, with Iranian and Saudi cohorts showing trends toward higher severity than the Egyptian cohorts, possibly reflecting differences in pre-hospital care infrastructure or referral pathways.

Clinical Outcomes and Complications

A pivotal finding of this review was the remarkably low in-ICU mortality rate (0%) and incidence of cerebral edema (<1%). It must be emphasized, however, that this mortality rate pertains strictly to patients who survived long enough to reach and be admitted to a tertiary intensive care setting. This underestimates the true regional mortality burden of DKA, as it does not capture pre-hospital deaths or patients who may have succumbed in emergency departments prior to ICU transfer. This favorable survival profile stands in stark contrast to historical global estimates that cite cerebral edema as the leading cause of DKA-related mortality [8]. This may reflect the high quality of specialized care provided in regional tertiary centers, where adherence to standardized fluid management protocols likely mitigates the risk of life-threatening cerebral injury. Nevertheless, the burden of morbidity remains significant as a high incidence of AKI (41.5%) was reported in one prospective Egyptian cohort [33]. While this figure cannot be generalized as a regional average, it serves as a critical signal of potential under-recognized morbidity. This underscores the need for regional centers to adopt standardized AKI diagnostic criteria, such as the Kidney Disease: Improving Global Outcomes (KDIGO) guidelines, and potentially explore DKA-specific renoprotective agents during the initial rehydration window to mitigate fluid-induced stress on the pediatric kidney [10].

Heterogeneity and Data Quality

Statistical heterogeneity was substantial (I^2^>75%) across most analyses, which is a common feature in meta-analyses of observational studies. The sensitivity analysis identified the study by Hegab et al. [33] as a key source of heterogeneity; its exclusion reduced I^2^ from 77% to 61%. This suggests that the prospective nature of that study, which employed more rigorous monitoring and strict diagnostic criteria for complications like AKI, provided a different clinical profile than the retrospective chart reviews. Such retrospective data may under-report morbidity due to inconsistent documentation or less stringent clinical observation in the medical record. The overall certainty of the evidence was graded as low due to this inconsistency and the observational nature of the included studies. Nevertheless, the risk of bias assessment indicated that the larger, more recent studies had high methodological quality, lending significant credibility to the main clinical findings.

Limitations

The geographic representation was limited to three countries (Saudi Arabia, Egypt, Iran), limiting the generalizability to the entire Middle Eastern region. The reliance on retrospective data in most studies introduces the potential for information bias and missing data, particularly regarding long-term outcomes such as neurocognitive function. In addition, the inability to pool length of stay data due to inconsistent reporting (means vs. medians) prevented a robust health economic analysis. Finally, we could not adjust for socioeconomic status or insurance coverage, which are factors known to influence DKA severity [9].

Implications for Practice and Policy

The high rate of severe DKA at presentation calls for intensified public health campaigns targeting early symptom recognition of T1DM in children. For clinicians, the significant risk of AKI highlights the need for careful fluid stewardship and renal monitoring beyond simple creatinine measurement. Future research in the region should prioritize prospective multicentre registries to standardize data collection and further elucidate the risk factors for AKI and long-term morbidity in this vulnerable population.

The high rate of AKI (41.5%) highlights a significant therapeutic 'white space'. Current management focuses on metabolic stabilization but may not address renal tubular oxidative stress and osmotic damage. Future research should explore DKA-specific renoprotective agents, such as Nrf2 activators or mitochondrial-targeted antioxidants, which could be administered during the initial 24-hour rehydration window to mitigate fluid-induced stress on the pediatric kidney.

## Conclusions

This systematic review highlights that while pediatric DKA management in Middle Eastern intensive care units achieves excellent survival rates with minimal neurological mortality, the region faces a significant burden of severe disease presentation. The high prevalence of severe acidosis and new-onset diabetes suggests missed opportunities for early community-based diagnosis and intervention. Also, the incidence of acute kidney injury, despite the low rate of cerebral edema, indicates a shifting of complications that requires refined fluid management strategies and vigilant renal monitoring. These findings underscore the need for standardized, region-specific protocols and public health initiatives aimed at earlier recognition of diabetes symptoms to reduce the intensity of critical care required for this population.
